# Epidemiology of extrapulmonary tuberculosis, Alameda County, California, 2010–2021

**DOI:** 10.1016/j.jctube.2026.100617

**Published:** 2026-05-12

**Authors:** Saliha Nasir, Rachel Marusinec, Amit S. Chitnis, Devan Jaganath

**Affiliations:** a Tuberculosis Section, Division of Communicable Disease Control and Prevention, Alameda County Health, Public Health Department, 1100 San Leandro Blvd, San Leandro, CA 94577, USA; b Division of Pediatric Infectious Diseases, University of California, San Francisco, 550 16th St., 4th Floor, San Francisco, CA 94158, USA; c Center for Tuberculosis, University of California, San Francisco, 550 16th St., 4th Floor, San Francisco, CA 94158 USA

**Keywords:** Extrapulmonary tuberculosis, Epidemiology, Public health, Surveillance

## Abstract

**Background:**

Extrapulmonary tuberculosis (EPTB) represents a range of disease manifestations, and is a major contributor to ongoing TB burden in the United States. We examined the incidence, trends and characteristics of EPTB in a high-burden TB county in Northern California.

**Methods:**

We extracted surveillance data for all TB cases in Alameda County during 2010–2021. TB was classified per national surveillance definitions, and EPTB included any site involvement other than the lung. We determined overall and annual incidence of EPTB in comparison to pulmonary TB (PTB), and assessed trends in incidence using Poisson regression and proportion with Joinpoint regression. We further compared clinical and demographic characteristics by TB site of disease.

**Results:**

Of 1,336 TB cases included, 372 (28%) had EPTB disease only. Lymph nodes were the most common site (38.7%), followed by pleura (17.7%), peritoneal (5.4%), and bone (4.8%). From 2010 to 2021, EPTB incidence decreased by 52% from 3.5 to 1.7 cases per 100,000. However, the proportion of TB cases that were extrapulmonary increased from 2015 to 2021 (8.3% annual change, 95% CI 5.2%–14.2%). In comparison to PTB, EPTB case-patients were more likely to be aged < 45 years old, have end-stage renal disease, and pyrazinamide monoresistance, but less likely to have microbiological confirmation.

**Conclusions:**

EPTB incidence has decreased over time, but the proportion of EPTB cases increased. Greater awareness of clinical and demographic characteristics of EPTB may guide targeted interventions to support TB elimination.

## Highlights

• The proportion of extrapulmonary TB (EPTB) cases is increasing in Alameda County.
• Over half of EPTB sites were lymph nodes and pleura; 4% involved the CNS.
• EPTB cases may be hard to diagnose as were less likely to have lab confirmation.
• Risk factors for EPTB were age < 45 years, renal disease, and South Asian descent.

## 1 Introduction

Tuberculosis (TB) remains an important clinical and public health problem. Although TB incidence rates in the United States (U.S.) have declined from 10.4 cases per 100,000 population to 2.2 cases per 100,000 population during 1992 to 2020, rates have started to increase since 2021, with the most recent TB rate reported as 3.0 cases per 100,000 population during 2024 [1], [2]. The increases in TB incidence suggest that intensified public health efforts are needed to eliminate TB disease in the U.S.

Although U.S. TB public health efforts are often focused on pulmonary TB (PTB) to reduce transmission, extrapulmonary TB (EPTB) disease remains an important issue. Since 1992, EPTB disease has accounted for approximately 20% of all reported TB cases [3], [4]. EPTB disease may present with a wide range of symptoms and can involve several organ systems including lymph nodes, bone, and central nervous system [5]. In addition, EPTB disease can be a challenge to diagnose as it may be difficult to obtain adequate specimens from clinical sites to confirm a TB diagnosis, which may lead to delays in starting patients on treatment [6].

Few studies have analyzed U.S. public health surveillance data to understand trends and epidemiology of EPTB disease [3], [7], [8], [9]. Alameda County is a high TB burden county in Northern California, with a 2024 TB incidence rate of 7.8 cases per 100,000 population, 1.4 times the California state rate and 2.6 times the national rate [10]. We utilized TB surveillance data from Alameda County during 2010–2021 to examine incidence and demographic and clinical characteristics of EPTB cases to guide public health interventions to reduce the burden of TB disease.

## 2 Methods

### 2.1 Setting

Alameda County is located in the eastern San Francisco Bay Area. The Alameda County Public Health Department (ACPHD) serves over 1.5 million residents of Alameda County. Of all Alameda County residents, 50% are female, 34% are non-Hispanic Asian, the largest racial ethnic category, and 33% are non-U.S. born [11].

### 2.2 Data sources

TB case information was extracted from Centers for Disease Control and Prevention (CDC) Report of Verified Case of Tuberculosis (RVCT) forms submitted by Alameda County for each case. A dataset of all Alameda County cases was provided to the county by the California Department of Public Health. We included all confirmed TB cases with PTB or EPTB disease reported to ACPHD during 2010–2021 and excluded cases with both PTB and EPTB disease to allow a clearer comparison based on presenting symptoms. Since this analysis of TB surveillance data was conducted to allow ACPHD to monitor, assess, and inform local TB public health interventions, no human subject review was required.

### 2.3 Definitions

For this analysis, we used the RVCT TB case definition [12]. The CDC RVCT defines a TB case as a patient who meets clinical (i.e., a positive skin or blood test for TB; signs and symptoms of TB disease; and treatment with two or more anti-TB medications; and a completed diagnostic evaluation) or laboratory criteria (i.e., isolation of *Mycobacterium tuberculosis* complex in a clinical specimen; or demonstration of *M. tuberculosis* complex from a clinical specimen by a nucleic acid amplification test (NAAT); or demonstration of acid-fast bacilli (AFB) in a clinical specimen when a culture has not been or cannot be obtained, is falsely negative, or contaminated) for TB diagnosis [12]. We defined TB site of disease according to the RVCT as extrapulmonary or pulmonary if the site of disease was in a location other than the lungs or in the lungs, respectively [12]. Our analysis included the case verification criteria that is calculated by the RVCT when classifying cases. This variable reports the main criterion that allowed the TB case to be considered a verified case [13]. We used the Healthy Places Index (HPI) to assess whether social and environmental-related health risk factors are associated with EPTB disease. HPI scores combine social factors conducive to health such as the environment of the neighborhood, education levels, and healthcare accessibility by census track and zip code [14]. For age category, we stratified by age < 45 years versus ≥ 45 years, as other studies have observed differences in EPTB risk with this cutoff [9], [15]. Other studies have found an association between EPTB and South Asian country of birth [15], and we stratified by this variable as well. For this analysis, we categorized South Asian countries based on a prior publication as Afghanistan, Bangladesh, Bhutan, India, Maldives, Nepal, Pakistan, and Sri Lanka [15].

### 2.4 Analysis

We calculated incidence rates per 100,000 population for EPTB and PTB cases, with the denominator based on Esri population estimates [16], [17]. We assessed trends in incidence rates for EPTB and PTB cases by using Poisson regression models; these models used annual case counts and the offset function to adjust for the log of the annual population of Alameda County residents. We evaluated trends in proportion of EPTB cases and 95% confidence intervals (95% CIs) using Joinpoint regression. The detection of time intervals during increasing or decreasing annual percent change (APC) among EPTB cases were identified by the Joinpoint software. We compared demographic and clinical characteristics of EPTB and PTB cases using Chi-square and Fisher’s exact test. For all analyses, we used SAS® 9.4 (SAS Institute, Inc., Cary, North Carolina), and Joinpoint software 5.3.0 (National Cancer Institute, Bethesda, Maryland).

## 3 Results

### 3.1 Trends in incidence and proportion of EPTB cases

**Fig. 1:**
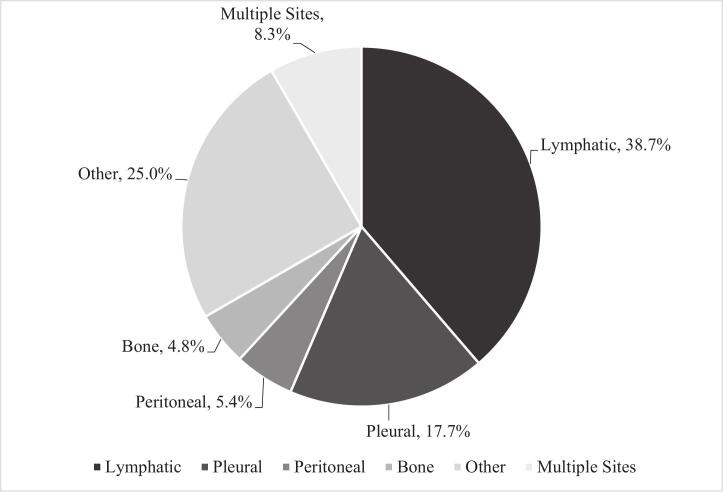
Distribution of anatomic sites of tuberculosis disease among extrapulmonary tuberculosis cases, Alameda County, 2010–2021. Extrapulmonary tuberculosis site categories were based on the Report for Verified Case of Tuberculosis. Other sites include Skin and Skin Appendages, Subcutaneous Tissue, Breast, Pericardium, Liver, Tonsils and Adenoids, Stomach, Small Intestine: Jejunum & Ileum, Colon, Brain, Spinal Cord, Cranial, Spinal, and Peripheral Nerve, Eye and Ear Appendages, Ear and Mastoid Cells.

During 2010–2021, a total of 1,532 TB cases were reported to Alameda County, of which 1,336 TB cases had extrapulmonary-only or pulmonary-only TB disease and were included in all analyses. Of all TB cases included in these analyses, 964 (72%) had PTB disease only, and 372 (28%) had EPTB disease only. Among EPTB cases, the most common anatomic site of disease were lymph nodes (38.7%), pleura (17.7%), peritoneal (5.4%), and bones (4.8%) (Fig. 1). Central nervous system disease was present in 14 (3.8%) cases.

**Fig. 2:**
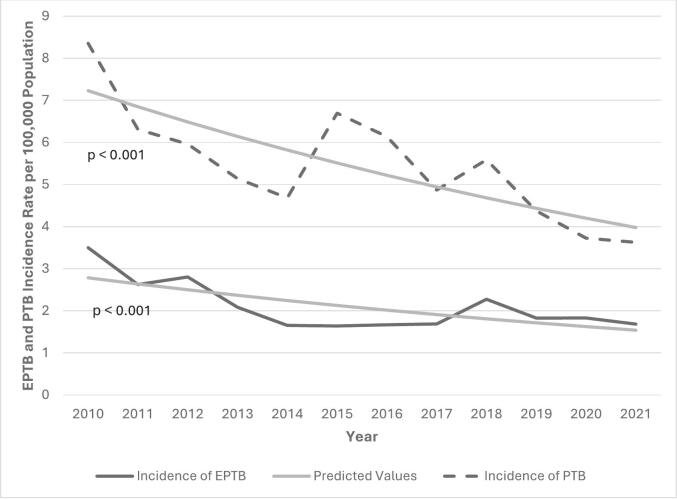
Trends in observed and predicted tuberculosis incidence rates per 100,000 population, by extrapulmonary and pulmonary site of disease only, Alameda County, 2010–2021. Predicted incidence rates were calculated by Poisson regression. Abbreviations: EPTB: extrapulmonary tuberculosis, PTB: pulmonary tuberculosis.

**Fig. 3:**
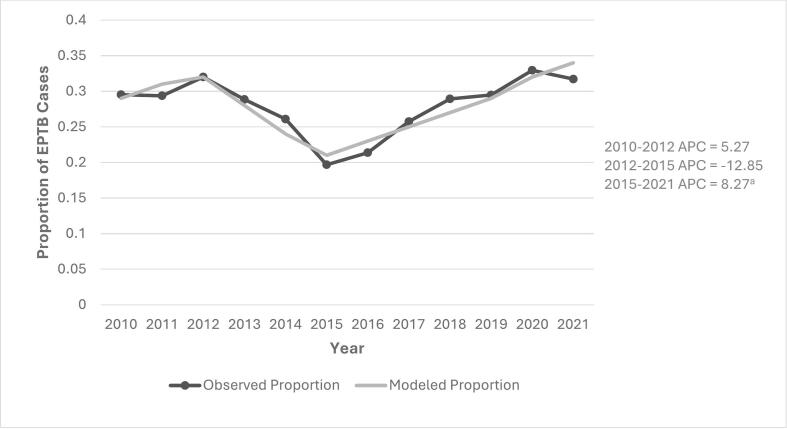
Trends in observed and modeled proportion of tuberculosis cases with extrapulmonary site of tuberculosis disease only, Alameda County, 2010–2021. Modeled proportion and APC trend determined by joinpoint regression analysis. Abbreviations: APC: annual percentage change, EPTB: extrapulmonary tuberculosis ^a^During 2015–2021, APC trend was statistically significant, p < 0.05.

The EPTB incidence rate significantly decreased by 52% from 3.5 cases per 100,000 population during 2010 to 1.7 cases per 100,000 population during 2021; the PTB incidence rate significantly decreased by 47% from 8.4 cases per 100,000 population during 2010 to 3.6 cases per 100,000 population during 2021 (both p < 0.001, Fig. 2). Joinpoint analysis of trends in proportion of EPTB cases detected three distinct time periods: non-significant increases in proportions during 2010–2012 (APC = 5.3%, 95% CI: −4.9%, 14.2%, Fig. 3); non-significant decreases in proportions during 2012–2015 (APC = -12.9%, 95% CI: −16.9%, 0.4%, Fig. 3); and a statistically significant increase in the proportions during 2015–2021 (APC = 8.3%, 95% CI: 5.2%, 14.2%, Fig. 3).

### 3.2 Comparison of demographic and clinical characteristics of extrapulmonary and pulmonary TB cases

Compared to PTB, case-patients with EPTB were more likely to be under 45 years old (45.8% vs 32.2%, p < 0.001), female (51.3% vs 37.7%, p > 0.001), and Hispanic (14.8% vs. 11.0%, p < 0.01); they were less likely to be Asian (66.4% vs. 72.6%, p < 0.01) [Table 1]. Among case-patients born in Asian countries, patients with EPTB were more likely to be from a South Asian country (37.8% vs. 19.9%, p < 0.001) than PTB case-patients. No significant differences in EPTB and PTB cases were detected by U.S. nativity, occupation, and HPI (Table 1).

**Table 1:** Comparison of demographic and social characteristics among tuberculosis cases, by extrapulmonary and pulmonary sites of disease, Alameda County, 2010–2021.

Characteristics	Extrapulmonary TB (N = 372)	Pulmonary TB (N = 964)	P-value^a^^,^^b^
***Demographic***			
Median age (IQR)	47 (33–63)	57 (36–73)	<0.001
Age group			<0.001
0–14 years old	14 (3.8%)	25 (2.6%)	
15–24 years old	26 (7.0%)	88 (9.1%)	
25–44 years old	130 (35.0%)	198 (20.5%)	
45–64 years old	118 (31.7%)	290 (30.1%)	
> 65 years old	84 (22.6%)	363 (37.7%)	
Sex			<0.001
Male	181 (48.7%)	601 (62.3%)	
Female	191 (51.3%)	363 (37.7%)	
Race/Ethnicity (%)			0.01
Hispanic	55 (14.8%)	106 (11.0%)	
Non-Hispanic white	18 (4.8%)	65 (6.7%)	
Non-Hispanic black	46 (12.4%)	73 (7.6%)	
Asian	247 (66.4%)	700 (72.6%)	
Other	6 (1.6%)	20 (2.1%)	
Country of birth			0.80
U.S.-born	49 (13.2%)	136 (14.1%)	
Non-U.S.-born	323 (86.8%)	827 (85.8%)	
Asian county of birth^c^			< 0.001
South Asian	90 (37.8%)	132 (19.9%)	
Other Asian	148 (62.2%)	532 (80.1%)	
Occupation^d^			0.77
Healthcare worker	19 (5.1%)	40 (4.2%)	
Non healthcare worker	352 (94.6%)	921 (95.5%)	
Unemployed^e^	57 (15.3%)	132 (13.7%)	0.78
***Social Risk Factors***			
Homeless in year prior to diagnosis	4 (1.1%)	19 (2.0%)	0.35
Resident of correctional facility at TB diagnosis	3 (0.8%)	15 (1.6%)	0.43
Alcohol use in past year	9 (2.4%)	44 (4.6%)	0.04
Healthy Places Index Quartile^f^			0.29
1st Quartile	132 (35.6%)	374 (38.9%)	
2nd Quartile	102 (27.5%)	283 (29.5%)	
3rd Quartile	92 (24.8%)	212 (22.1%)	
4th Quartile	45 (12.1%)	92 (9.6%)	

Abbreviations: TB: tuberculosis.

a p-value: Chi-square or Fisher’s exact test was conducted to compare categorical variables.

b Kruskal-Wallis rank sum test was used to calculate p-value for continuous variables.

c South Asian includes: Afghanistan, Bangladesh, Bhutan, India, Maldives, Nepal, Pakistan, and Sri Lanka individuals. Other Asian individuals include individuals from Cambodia, China, East Timor, Fiji, Indonesia, Kiribati, North Korea, Laos, Malaysia, Marshall Islands, Micronesia, Mongolia, Myanmar, Palau, Papua New Guinea, Philippines, Samoa, Solomon Islands, South Korea, Thailand, Tonga, Tuvalu, Vanuatu, and Vietnam.

d Refers to primary role within the past year according to RVCT [12].

e Refers to primary occupation within the past year listed as the unemployed category in RVCT [12].

f An index measuring factors conducive to health by geographic location [14]. Locations in higher quartiles are more conducive to health. Cases missing address information were excluded from denominator.

With regards to clinical characteristics, EPTB case-patients were significantly less likely than PTB case-patients to have diabetes (13.2% vs. 27.9%, p < 0.01), a positive culture from any anatomic site (60.8% vs. 81.4%, p < 0.001), be verified as a TB case by laboratory criteria (63.4% vs. 83.0%, p < 0.001), and die before or during treatment (2.7% vs. 9.4%, p < 0.001); they were significantly more likely to have end-stage renal disease (5.4% vs. 2.1%, p < 0.01), and pyrazinamide-monoresistance (6.4% vs. 1.6%, p < 0.001) (Table 2). Five of the twelve (42%) genotyped pyrazinamide-monoresistant EPTB case-patients had *M. bovis*. The remaining seven case-patients’ isolates were *M. tuberculosis*. Of these seven, five had a lineage identified – all were Indo-Oceanic. Among non-*M. bovis* cases, EPTB cases were still more likely to have pyrazinamide-monoresistance than PTB cases (4.1% vs. 1.5%, p = 0.04).

**Table 2:** Comparison of clinical characteristics, laboratory, and outcomes of tuberculosis cases, by extrapulmonary and pulmonary sites of disease, Alameda County, 2010–2021.

Characteristics	Extrapulmonary TB (N = 372)	Pulmonary TB (N = 964)	P-value^a^
***Clinical***			
Diabetes	49 (13.2%)	269 (27.9%)	<0.001
End stage renal disease	20 (5.4%)	20 (2.1%)	<0.01
HIV status^b^			0.71
Positive	7 (1.9%)	23 (2.4%)	
Negative	253 (68.0%)	676 (70.1%)	
Refused	12 (3.2%)	29 (3.0%)	
Not offered	36 (9.7%)	72 (7.5%)	
Unknown	64 (17.2%)	164 (17.0%)	
Non-HIV immunocompromised	21 (5.7%)	58 (6.0%)	0.80
***Laboratory***			
IGRA			0.02
Positive	187 (50.3%)	409 (42.4%)	
Negative	39 (10.5%)	93 (9.7%)	
Not done	130 (35.0%)	420 (43.6%)	
Indeterminate	14 (3.8%)	26 (2.7%)	
Unknown	2 (0.5%)	16 (1.7%)	
TST			0.96
Positive	91 (24.5%)	242 (25.1%)	
Negative	16 (4.3%)	45 (4.7%)	
Not done	251 (67.5%)	637 (66.1%)	
Unknown	14 (3.8%)	40 (4.2%)	
NAAT			
Positive	48 (12.9%)	395 (41.0%)	<0.001
Negative	54 (14.5%)	72 (7.5%)	
Not Done	268 (72.0%)	497 (51.6%)	
Indeterminate	2 (0.5%)	0 (0%)	
Positive culture (all anatomic sites)	226 (60.8%)	785 (81.4%)	<0.001
Sputum culture			<0.001
Positive culture	0 (0%)	707 (73.3%)	
Negative culture	302 (81.2%)	208 (21.6%)	
Not done	63 (16.9%)	43 (4.5%)	
Unknown	7 (1.9%)	6 (0.6%)	
Culture of tissue and other body fluids^c^			<0.001
Positive culture	226 (60.8%)	161 (16.7%)	
Negative culture	92 (24.7%)	88 (9.1%)	
Not done	53 (14.3%)	710 (73.7%)	
Unknown	1 (0.3%)	5 (0.5%)	
Positive AFB stain or pathology/cytology consistent with TB^c^			<0.001
Positive Smear or pathology/cytology	87 (23.4%)	85 (8.8%)	
Both positive	25 (6.7%)	18 (1.9%)	
Smear only positive	34 (9.1%)	48 (5.0%)	
Pathology/cytology only positive	28 (7.5%)	17 (1.8%)	
Both negative	252 (67.7%)	184 (19.1%)	
Not done	33 (8.9%)	695 (72.1%)	
Drug resistance			
Multidrug-resistant^d^	2 (0.9%)	13 (1.7%)	0.54
Isoniazid monoresistant^e^	15 (7.4%)	86 (11.5%)	0.10
Pyrazinamide monoresistant^f^	13 (6.4%)	11 (1.6%)	<0.001
RVCT Case Verification Criteria			<0.001
Laboratory-confirmed	236 (63.4%)	800 (83.0%)	
Positive culture	226 (60.8%)	785 (81.4%)	
Positive NAA test, culture negative or not done	9 (2.4%)	15 (1.6%)	
Positive smear/tissue	1 (0.3%)	0 (0%)	
Clinically-confirmed	99 (26.6%)	121 (12.6%)	
Verified by Provider Diagnosis	37 (10.0%)	43 (4.5%)	
***Outcomes***			
Treatment modality			<0.001
Self-administered therapy	184 (49.5%)	140 (14.5%)	
Directly observed therapy	120 (32.3%)	587 (60.9%)	
Both	59 (15.9%)	211 (21.9%)	
Unknown	9 (2.4%)	26 (2.7%)	
Died before or during treatment	10 (2.7%)	91 (9.4%)	<0.001

Abbreviations: HIV: human immunodeficiency virus; IGRA: interferon gamma-release assay; MDR: multidrug- resistant TB; TB: tuberculosis; TST: tuberculin skin test.

a Chi-square or Fisher’s Exact Test was conducted to compare categorical variables.

b HIV status at the time of diagnosis according to RVCT[12].

c Does not include sputum. Examples of tissues and other body fluids for pulmonary cases include bronchial cells and fluid, bronchoalveolar lavage fluid, gastric aspirate, and lung tissue/fluid. Includes samples of other body sites for extrapulmonary TB according to RVCT[12].

d MDR: phenotypic drug testing resistant to both rifampin and isoniazid. Missing, unknown, or laboratory exams listed as not done were excluded from denominator for the Fisher’s exact test.

e Isoniazid monoresistance: phenotypic drug testing resistant to the isoniazid and known susceptibility to rifampin, ethambutol, and pyrazinamide. Missing, unknown, or laboratory exams listed as not done were excluded from denominator for the Fisher’s exact test.

f Pyrazinamide monoresistance: phenotypic drug testing resistant to pyrazinamide, and known susceptibility to isoniazid, rifampin, and ethambutol. Missing, unknown, or laboratory exams listed as not done were excluded from denominator for the Fisher’s exact test.

### 3.3 Characteristics of extrapulmonary and pulmonary TB cases stratified by age

EPTB and PTB case-patients were stratified by age category (<45 vs. ≥ 45 years old) to determine if there were any differences in demographic characteristics (Supplementary Fig. 1). Among TB case-patients under 45 years of age, there were no statistically significant differences between EPTB and PTB case-patients by sex, race/ethnicity categories, and death before or during TB treatment. However, EPTB case-patients were significantly more likely to be born in South Asia (36.5% vs. 19.3%, p < 0.01). Among TB case-patients aged 45 years and older, EPTB patients were significantly more likely to be Hispanic (11.9% vs. 7.4%, p = 0.02), from a non-Asian birth country (30.7% vs. 23.3%, p = 0.03), and female (50.0% vs. 33.8%, p < 0.001); they were less likely to have died before or during TB treatment (5.0% vs. 13.5%, p < 0.001).

## 4 Discussion

EPTB disease remains an important clinical and public health problem, and accounted for 28% of TB cases in Alameda County during 2010–2021. While incidence of EPTB disease decreased during this time period, the proportion of TB cases that were EPTB disease increased during 2015–2021. We found EPTB, compared to PTB, case-patients more likely to be younger (<45 years old), female, to have end-stage renal disease, and be diagnosed by clinical criteria. Greater recognition and screening for EPTB is needed to reduce the national burden of TB disease.

Our analyses found that the incidence of EPTB and PTB significantly decreased during 2010–2021, consistent with the trend nationally and in similar settings [3], [18], [19]. At the same time, the proportion of TB cases that were EPTB increased significantly, and this was higher than national data (8.27% vs. 1.61% during 2016–2021) [3]. Other low-incidence settings have had mixed findings, with an increase in EPTB proportion in the Netherlands during 1993–2022 [20], but no significant difference in Spain during this time period [21]. While settings in Europe have related the increase of EPTB to an increase in immigration [20], [22], our analysis did not find a significant difference between U.S.-born and non-U.S.-born case-patients. The increase in the proportion of EPTB in our county may be due, in part, to the decrease in PTB incidence due to improved screening and contact investigation efforts. This suggests that while current TB control efforts are having an effect on PTB incidence, more attention should be brought to EPTB case identification and prevention. Our findings can inform these next steps, by increasing awareness and knowledge of the main presentations of EPTB, and ensuring that populations at higher risk of EPTB are appropriately screened and treated for TB infection.

While screening and identifying PTB may have improved during this time, diagnostic workup difficulties may have continued for EPTB cases. The most common EPTB disease sites in Alameda County were lymph nodes, followed by pleura, as seen in other low-incidence TB settings [9], [18], [19]. Diagnoses for these cases can be challenging, with non-specific findings. In addition, these sites often require further intervention for diagnosis, including fine needle aspiration, biopsy, and thoracentesis, which may not be routinely available at lower-level facilities. When compared to PTB, EPTB cases in our county were more often verified by meeting the clinical case definition, and less often by a positive laboratory test. Culture-confirmed EPTB cases were dependent on sampling from tissue and bodily fluids, which may be difficult to obtain and lead to delays or missed diagnosis.

Another possibility for the increase in EPTB proportion may be a larger impact of the COVID-19 pandemic on PTB incidence than EPTB incidence due to reduced transmission during the lockdown. However, the limited studies that examined this relationship have had mixed results; one study in Hong Kong noted an overall decline in EPTB proportion during the COVID-19 pandemic, while a study in Spain reported a decrease in PTB diagnoses and increase in EPTB diagnoses during the pandemic [23], [24]. In addition, due to changes to the RVCT form introduced in 2022, our analysis only went through the first two years of the pandemic; with the recent rise in TB cases since 2021, additional research is needed to examine how EPTB trends have further changed.

Our analyses found female sex and South-Asian nativity to be associated with EPTB, as noted in prior studies [18], [25], [26]. A study of EPTB case-patients in the U.S. by Asghar et al. found no relationship between common EPTB risk factors (sex, HIV status, *M. bovis* infection, diagnosis methods) and South-Asian ethnicity when trying to determine why EPTB was more prevalent in this ethnic group [27]. Another study by Stennis et al. examined TB cases among South Asian patients in New York and did not find social or clinical characteristics explaining the high burden among this group [15]. It is speculated that there is an unknown genetic background or physiological response to TB among South Asians that may increase risk of extrapulmonary complications [27]. Moreover, there is growing literature on the association between the L1 (Indo-Oceanic) lineage, which is mainly found in South and Southeast Asia and East Africa, and EPTB [28]. One reason postulated for EPTB being more common among females is that smoking, a risk factor associated with PTB, is more common among men [25]. However, our dataset did not include a smoking variable for the years included in this analysis, and so could not be used to test for confounding. Another hypothesis for the ETPB-female sex association is the result of endocrine-related factors, as studies have found EPTB more common among older women of post-menopause age [29], an association we also found when stratifying case-patients to age groups over and under 45 years of age. Pregnancy was not recorded in the RVCT form, and we were unable to assess if pregnancy also may influence risk of EPTB.

Among clinical risk factors for TB, diabetes and end-stage renal disease were found to have a negative and positive association with EPTB, respectively. Diabetes, a known risk factor for TB, was less common among EPTB, compared to PTB, case-patients, and was consistent with that of other studies in both high- and low- incidence settings which have found no or negative association for diabetes and EPTB-only case-patients compared to PTB case-patients [20], [30], [31]. Conversely, end-stage renal disease, another known risk factor for TB [32], was significantly more common among EPTB, compared to PTB, case-patients in our analysis, a trend that has also been observed in other U.S. populations [30], [31]. It is thought that kidney damage, as well as the reduced immune response due to dialysis or immune suppression after transplant, could cause progression from LTBI to EPTB, as well as leaving patients more susceptible to *M. bovis* [9], [31]. This highlights the need to adhere to screening guidelines for patients with renal disease [33]. In contrast to other studies [30], [34], our analysis found no association between HIV and EPTB. However, case counts of HIV were low among our population and may not have had the statistical power to observe an association. While the case-patient characteristics described above are established risk factors for TB, EPTB in particular should be considered for patients belonging to populations with these clinical and demographic risk factors, especially those that are immunosuppressed, when presenting with symptoms that may not indicate PTB.

Our analysis found that two-thirds of PTB case-patients were over 45 years of age, whereas that same age group only made up around half of EPTB case-patients. Other studies have found EPTB to be more common among younger patients especially when compared to the 45–64 age group, a finding we also observed [9], [25], [35]. We stratified the cases by over and under 45 years of age to determine differences in characteristics. EPTB case-patients were significantly more likely to be South Asian than PTB case-patients in both age groups, but the proportion of EPTB case-patients that were South Asian was much higher among the younger EPTB patients. Given that South Asian nativity is associated with EPTB [15], [25], this could be part of the reason EPTB was more common among this younger age group.

Our findings showed that pyrazinamide monoresistance was four times higher among EPTB cases than PTB cases. Multiple studies have shown an association between pyrazinamide monoresistance and EPTB [36], [37]. *M. bovis* has been found to be associated with pyrazinamide resistance [37], as well as EPTB [38], and made up a large proportion of pyrazinamide-monoresistant EPTB cases in our analysis. While *M. bovis* may account for some of the association seen between pyrazinamide monoresistance and EPTB, the association was still significant when examined among non-*M. bovis* cases. Other studies have noted an association between pyrazinamide monoresistance and other *M. tuberculosis* lineages such as the Indo-Oceanic lineage [37], which may also have an association with EPTB [28]. Five of the seven genotyped non-*M. bovis* pyrazinamide monoresistant cases in our data had a lineage identified – all were Indo-Oceanic. For clinicians and public health practitioners, these findings are important when considering EPTB for two reasons. First, they highlight the importance of obtaining clinical specimens to confirm a microbiologic diagnosis of TB and for obtaining phenotypic or molecular drug susceptibility tests, as detection of pyrazinamide resistance results in a 9-month rather than 6-month treatment duration [39]. Second, because EPTB case-patients are less likely to have a positive culture, it is important to ask patients if they have risk factors for *M. bovis* such as consuming unpasteurized dairy products or whether they have epidemiologic risk factors for *M. bovis* such as Hispanic ethnicity, HIV, or diabetes [40].

This analysis is subject to limitations. We were limited to the information collected on the RVCT form and did not have the ability to access medical chart information; other factors that may have been associated with TB, such as smoking status, pregnancy, and antiretroviral therapy status, could not be explored. While we analyzed a twelve-year period, our analysis focused on one county, and there may be limited generalizability. However, Alameda County is high-burden compared to other U.S. jurisdictions, and the findings may be applicable to other diverse, urban counties in the U.S. Due to changes in the RVCT form introduced in 2022, our analysis did not extend beyond the first two years of the COVID-19 pandemic, and trends in proportion of EPTB may change now that TB cases are once again increasing in the U.S, a topic for further study.

In conclusion, we noted while incidence rates of both PTB and EPTB declined during 2010–2021, the proportion of cases that were EPTB increased from 2015 to 2021. Risk factors associated with EPTB include being female, of South-Asian nativity, aged < 45 years old, and having end-stage-renal disease. The lymphatic system was the most common site of EPTB, and EPTB cases were more often confirmed clinically than with laboratory tests compared to PTB cases, highlighting that diagnosing of EPTB remains difficult. Greater awareness of clinical and demographic characteristics of EPTB among clinicians and public health practitioners may be needed to guide targeted interventions to support the elimination of TB.

## Ethics Statement

This analysis and collection of TB surveillance data were performed as part of ACPHD’s public health activities and purposes to conduct surveillance, assess, and inform local public health interventions; thus no human subject review was required in accordance with U.S. Code of Federal Regulations, 45 CFR 46.101.

## CRediT authorship contribution statement

**Saliha Nasir:** Writing – original draft, Visualization, Formal analysis. **Rachel Marusinec:** Writing – review & editing, Writing – original draft, Supervision. **Amit S. Chitnis:** Writing – review & editing, Supervision, Conceptualization. **Devan Jaganath:** Writing – review & editing, Conceptualization.

## Funding

This research did not receive any specific funding from the public, commercial, or not-for-profit sectors. DJ receives support from the National Institutes of Health (R01HL169449, R01AI90419).

## Declaration of competing interest

The authors declare that they have no known competing financial interests or personal relationships that could have appeared to influence the work reported in this paper.
