# Supplementary material for: Epidemiology of extrapulmonary tuberculosis, Alameda County, California, 2010–2021
Source: J Clin Tuberc Other Mycobact Dis. 2026 May 12;44:100617. doi: 10.1016/j.jctube.2026.100617 (PMC13272544; doi:10.1016/j.jctube.2026.100617)
Supplement: Supplementary Data 1 [file mmc1.docx]

**Supplementary Table 1**. Comparison of demographic characteristics and outcomes among extrapulmonary and pulmonary tuberculosis cases, stratified by age, Alameda County, 2010–2021

| **Characteristics** | **Aged < 45 years old**  **(n=481)** | | **p-value^a^** | **Aged ≥ 45 years old**  **(n=855)** | | **p-value^a^** |
| --- | --- | --- | --- | --- | --- | --- |
|  | Extrapulmonary TB  (n=170, 35.3%) | Pulmonary  TB  (n=311, 64.7%) |  | Extrapulmonary TB  (n=202, 23.6%) | Pulmonary TB  (n=653, 76.4%) |  |
| Race/Ethnicity |  |  | 0.47 |  |  | 0.02 |
| Hispanic | 31 (18.2%) | 58 (18.7%) |  | 24 (11.9%) | 48 (7.4%) |  |
| Non-Hispanic White | 7 (4.1%) | 19 (6.1%) |  | 11 (5.5%) | 46 (7.0%) |  |
| Non-Hispanic Black | 24 (14.1%) | 35 (11.3%) |  | 22 (10.9%) | 38 (5.8%) |  |
| Asian | 105 (61.8%) | 186 (59.8%) |  | 142 (70.3%) | 514 (78.7%) |  |
| Other | 3 (1.8%) | 13 (4.2%) |  | 3 (1.5%) | 7 (1.1%) |  |
| Country of birth^b^ |  |  | <0.01 |  |  | 0.03 |
| South Asian | 62 (36.5%) | 60 (19.3%) |  | 28 (13.9%) | 72 (11.0%) |  |
| Other Asian | 36 (21.2%) | 103 (33.1%) |  | 112 (55.5%) | 429  (65.7%) |  |
| Non-Asian | 72 (42.4%) | 148 (47.6%) |  | 62 (30.7%) | 152 (23.3%) |  |
| Sex |  |  | 0.13 |  |  | <0.001 |
| Male | 80 (47.1%) | 169 (54.3%) |  | 101 (50.0%) | 432 (66.2%) |  |
| Female | 90 (53.0%) | 142 (45.7%) |  | 101 (50.0%) | 221 (33.8%) |  |
| Died Before or During Treatment | 0 (0%) | 3 (1.0%) | 0.56 | 10 (5.0%) | 88 (13.5%) | <0.001 |

TB: tuberculosis

^a^p-value: Chi-square or Fisher’s exact test was conducted to compare categorical variables

^b^South Asian birth country includes: Afghanistan, Bangladesh, Bhutan, India, Maldives, Nepal, Pakistan, and Sri Lanka individuals. Other Asian individuals include individuals from Cambodia, China, East Timor, Fiji, Indonesia, Kiribati, North Korea, Laos, Malaysia, Marshall Islands, Micronesia, Mongolia, Myanmar, Palau, Papua New Guinea, Philippines, Samoa, Solomon Islands, South Korea, Thailand, Tonga, Tuvalu, Vanuatu, and Vietnam. There were two missing country of birth classifications for pulmonary TB cases
